# Development and validation of a risk prediction nomogram for in-stent restenosis in patients undergoing percutaneous coronary intervention

**DOI:** 10.1186/s12872-021-02255-4

**Published:** 2021-09-14

**Authors:** Wenbo He, Changwu Xu, Xiaoying Wang, Jiyong Lei, Qinfang Qiu, Yingying Hu, Da Luo

**Affiliations:** 1 Department of Cardiology, Renmin Hospital of Wuhan University, 238 Jiefang Road, Wuchang, Wuhan, 430060 People’s Republic of China; 2 Cardiovascular Research Institute of Wuhan University, Wuhan, China; 3 Hubei Key Laboratory of Cardiology, Wuhan, China; 4 Department of Cardiology, Zhongnan Hospital of Wuhan University, Wuhan, China

**Keywords:** Predictors, Nomogram, Percutaneous coronary intervention, In-stent restenosis

## Abstract

**Background:**

This study aimed to develop and validate a nomogram to predict probability of in-stent restenosis (ISR) in patients undergoing percutaneous coronary intervention (PCI).

**Methods:**

Patients undergoing PCI with drug-eluting stents between July 2009 and August 2011 were retrieved from a cohort study in a high-volume PCI center, and further randomly assigned to training and validation sets. The least absolute shrinkage and selection operator (LASSO) regression model was used to screen out significant features for construction of nomogram. Multivariable logistic regression analysis was applied to build a nomogram-based predicting model incorporating the variables selected in the LASSO regression model. The area under the curve (AUC) of the receiver operating characteristics (ROC), calibration plot and decision curve analysis (DCA) were performed to estimate the discrimination, calibration and utility of the nomogram model respectively.

**Results:**

A total of 463 patients with DES implantation were enrolled and randomized in the development and validation sets. The predication nomogram was constructed with five risk factors including prior PCI, hyperglycemia, stents in left anterior descending artery (LAD), stent type, and absence of clopidogrel, which proved reliable for quantifying risks of ISR for patients with stent implantation. The AUC of development and validation set were 0.706 and 0.662, respectively, indicating that the prediction model displayed moderate discrimination capacity to predict restenosis. The high quality of calibration plots in both datasets demonstrated strong concordance performance of the nomogram model. Moreover, DCA showed that the nomogram was clinically useful when intervention was decided at the possibility threshold of 9%, indicating good utility for clinical decision-making.

**Conclusions:**

The individualized prediction nomogram incorporating 5 commonly clinical and angiographic characteristics for patients undergoing PCI can be conveniently used to facilitate early identification and improved screening of patients at higher risk of ISR.

## Background

Coronary heart disease severely threatens the health of individuals worldwide with a rising incidence and a leading cause of mortality. Revascularization with percutaneous coronary intervention (PCI) is a well-established and effective therapeutic strategy for advanced coronary heart disease, particularly following the introduction of drug-eluting stents (DES) [1]. While the incidence of in-stent restenosis (ISR) and target lesion revascularization is substantially reduced by DES compared with the bare-metal stents (BMS), they are not eliminated. ISR after DES implantation with an incidence of 3–20% remains a pervasive clinical problem which should not be neglected [2–4]. Hence, tools for the identification of individual patients at higher risk for ISR with stent implantation are especially needed.

Although several prior studies have analyzed potential predictive factors associated with a high incidence of ISR based on patient and procedure-related factors, there are still some limitations that confine their clinical application. Prediction model for ISR is yet to be fully developed and validated [5–7]. Of note, most studies have only focused on comprehensively identifying the predictors of ISR or developing prediction models without an individual risk prediction tool, while the simplicity and ease of use for the clinicians and patients were not well considered [7–9]. Thus, a more solid, well-validated and easy-to-use clinical ISR prediction model for all patients with stent implantation, especially for clinical decision in primary prevention, is urgently needed for accurate prognostication of future events.

The nomogram-based predicting model has been widely implemented in clinical studies. Featured by the advantage of visualization, a well-developed nomogram based on statistical regression models is a cogent tool to make clinical decision for clinicians and to assess straightforward the probability of disease for individual patients without complex formula, thus could benefit both doctors and patients.

Based on these premises, this study aimed to identify factors correlated to the risk of ISR for individual patients undergoing PCI, using data from an observational single-center registry study. These factors were used to develop and validate a nomogram-based clinical prediction model. This model could help clinicians discern high-risk ISR patients, optimize treatment strategy, and improve prognosis of these patients.

## Methods

### Study population

This study was a secondary analysis of an observational cohort study conducted between July 2009 and August 2011, in a high-volume PCI center, in Henan Province, China [10]. A total of 2,533 patients undergoing PCI with DES were enrolled and the median follow-up time was 29.8 months. The protocol was approved by the ethical committee in accordance with local regulations. The original work was in accordance with the Creative Commons Attribution Non-Commercial (CC BY-NC 3.0) license. The datasets analyzed during the current study are available in the Dryad data repository, https://doi.org/10.5061/dryad.13d31 [11]. Dyrad is a non-profit membership organization that is committed to making data available for research and educational reuse. Informed consent was waived because this is a post-hoc study using existing research data.

**Fig. 1:**
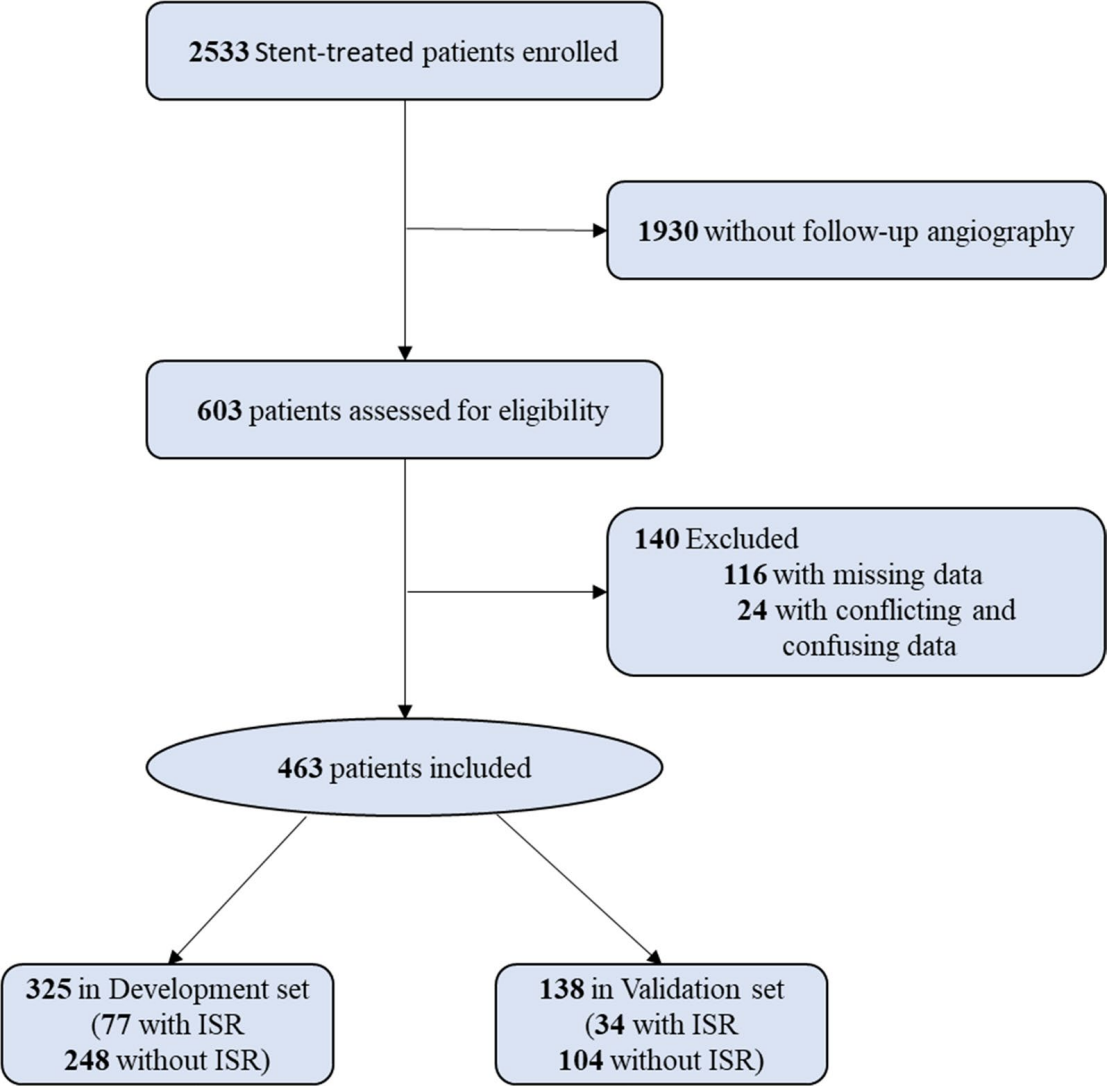
Study flow diagram for developing and validating the ISR risk model

The goal of this study was to identify factors associated with ISR in the patients with DES implantation, considering individual patient characteristics and their independent connection with ISR events. Generally, binary angiographic restenosis is defined as ≥ 50% luminal narrowing at follow-up angiography. Thus, we excluded 1,930 patients who were lost to angiographic follow-up and 116 patients with missing data. In addition, 24 patients with > 1 type of stent but the location of stents unknown were also screened out. After these exclusions, 463 patients were at last randomized and analyzed (Fig. 1).

### Risk factors

A total of 36 candidate variables, based on clinical plausibility and previous studies, were identified for further processing. The variables considered in this study were clinical characteristics (age, gender, presentation, old myocardial infarction, prior PCI, heart failure, atrial fibrillation, hypertension, diabetes mellitus, smoking), laboratory tests (systolic BP, diastolic BP, glycemia, uric acid, LDL-C), lesion and procedure characteristics (acute occlusion lesions, chronic total occlusions, restenotic lesions, location of stents, number of treated vessels, stent type, stent number, total stent length, stent diameter), and use of medications (aspirin, clopidogrel, statins, β-receptor blockers, ACEIs/ARBs, calcium channel blockers).

### Statistical analysis

To facilitate ease of use, all independent variables were transformed into categorical variables and expressed as count (%) based on confirmation that the gradient of effect was maintained. Statistical analysis was performed using the R software version 3.6.3, and two-tailed analysis with *P* < 0.05 was considered statistically significant.

We randomly designated 70% of the study population as the development set, while the rest were divided into the validation set. Patients in the development and validation set were compared using Chi-square test. The LASSO-penalized regression analysis, which is competent to estimate the parameters in high-dimensional regression, was applied to select predictors of ISR using the R package Glmnet [12, 13]. After the ISR predictors were confirmed, multivariable logistic regression analysis was used in the development set to evaluate the predictors and construct a nomogram predicting model along with their associated odds ratios (OR), 95% confidence intervals (CI), β-coefficients, and *P*-value.

The nomogram model performance was evaluated in terms of discrimination and calibration. The discrimination of the model was assessed by calculating the area under the curve (AUC) of the receiver operating characteristics (ROC), which indicated the predictive accuracy of the nomogram. Generally, the diagnostic accuracy with an AUC equal or above 0.6 is considered acceptable [14, 15]. Calibration plots were used for the comparison between the predicted and observed probabilities in both sets, and a significant difference implies the poor calibration of the prediction model. Furthermore, decision curve analysis (DCA) plotted net benefit (NB) at a range of clinically reasonable risk thresholds, helping assess the clinical usefulness of the model for decision making [16].

## Results

### Patient characteristics

A total of 463 patients undergoing PCI with DES were enrolled and randomized in this study, including 325 in the development set and 138 in the validation set. Table 1 lists 36 variables including the following aspects: clinical characteristics, laboratory findings, lesion and procedure characteristics, and use of medications for all randomized patients. Of these, 111 patients (77 in the development set and 34 in the validation set) developed in-stent restenosis. Comparisons between the development and validation sets showed no significant differences in all ISR risk-related variables (all *P* > 0.05).

**Table 1:** Baseline characteristics of the development set and validation set

Measure	Development set	Validation set	*P* value
	(n = 325)	(n = 138)	
*Clinical characteristics*			
Age, year, n (%)			0.579
< 50	78 (24.0)	27 (19.6)	
50–70	195 (60.0)	88 (63.8)	
≥ 70	52 (16.0)	23 (16.7)	
Gender, n (%)			0.282
Female	104 (32.0)	52 (37.7)	
Male	221 (68.0)	86 (62.3)	
Presentation, n (%)			0.415
STEMI with Urgent PCI	12 (3.7)	3 (2.2)	
STEMI with Delayed PCI	59 (18.2)	20 (14.5)	
NSTE-ACS	198 (60.9)	95 (68.8)	
SA	56 (17.2)	20 (14.5)	
OMI, n (%)			0.381
No	294 (90.5)	129 (93.5)	
Yes	31 (9.5)	9 (6.5)	
Prior PCI, n (%)			0.616
No	294 (90.5)	122 (88.4)	
Yes	31 (9.5)	16 (11.6)	
Heart failure, n (%)			1
No	286 (88.0)	121 (87.7)	
Yes	39 (12.0)	17 (12.3)	
Atrial fibrillation, n (%)			0.288
No	320 (98.5)	133 (96.4)	
Yes	5 (1.5)	5 (3.6)	
Hypertension, n (%)			0.664
No	169 (52.0)	68 (49.3)	
Yes	156 (48.0)	70 (50.7)	
Diabetes mellitus, n (%)			0.815
No	259 (79.7)	112 (81.2)	
Yes	66 (20.3)	26 (18.8)	
Smoke, n (%)			0.496
No	209 (64.3)	94 (68.1)	
Yes	116 (35.7)	44 (31.9)	
*Laboratory tests*			
Systolic BP, n (%)			0.749
< 90 mmHg	92 (28.3)	44 (31.9)	
90–140 mmHg	181 (55.7)	68 (49.3)	
140–160 mmHg	40 (12.3)	20 (14.5)	
160–180 mmHg	11 (3.4)	5 (3.6)	
≥ 180 mmHg	1 (0.3)	1 (0.7)	
Diastolic BP, n (%)			0.76
< 60 mmHg	18 (5.5)	6 (4.3)	
60–80 mmHg	143 (44.0)	65 (47.1)	
80–100 mmHg	150 (46.2)	59 (42.8)	
≥ 100 mmHg	14 (4.3)	8 (5.8)	
Glycemia, n (%)			0.837
< 6.1 mmol/L	237 (72.9)	104 (75.4)	
6.1–11.1 mmol/L	79 (24.3)	30 (21.7)	
≥ 11.1 mmol/L	9 (2.8)	4 (2.9)	
Creatinine, n (%)			0.321
< 110 mmol/L	313 (96.3)	136 (98.6)	
≥ 110 mmol/L	12 (3.7)	2 (1.4)	
Uric Acid, n (%)			1
< 400 µmol/L	283 (87.1)	120 (87.0)	
≥ 400 µmol/L	42 (12.9)	18 (13.0)	
LDL-C, n (%)			0.06
< 1.80 mmol/L	66 (20.3)	17 (12.3)	
1.80–3.63 mmol/L	220 (67.7)	96 (69.6)	
3.63–4.14 mmol/L	24 (7.4)	12 (8.7)	
≥ 4.14 mmol/L	15 (4.6)	13 (9.4)	
*Lesion and procedure characteristics*			
Acute occlusion lesions, n (%)			0.645
No	283 (87.1)	123 (89.1)	
Yes	42 (12.9)	15 (10.9)	
Chronic total occlusions, n (%)			0.157
No	294 (90.5)	131 (94.9)	
Yes	31 (9.5)	7 (5.1)	
Ostial lesions, n (%)			0.646
No	288 (88.6)	125 (90.6)	
Yes	37 (11.4)	13 (9.4)	
Bifurcation lesions, n (%)			0.676
No	257 (79.1)	106 (76.8)	
Yes	68 (20.9)	32 (23.2)	
Restenotic lesions, n (%)			0.603
No	316 (97.2)	136 (98.6)	
Yes	9 (2.8)	2 (1.4)	
Location of stents, n (%)			
LM, n (%)			1
No	312 (96.0)	132 (95.7)	
Yes	13 (4.0)	6 (4.3)	
LAD, n (%)			0.56
No	84 (25.8)	40 (29.0)	
Yes	241 (74.2)	98 (71.0)	
LCX, n (%)			0.413
No	187 (57.5)	73 (52.9)	
Yes	138 (42.5)	65 (47.1)	
RCA, n (%)			1
No	198 (60.9)	84 (60.9)	
Yes	127 (39.1)	54 (39.1)	
Number of treated vessels, n (%)			0.946
1	177 (54.5)	73 (52.9)	
2	106 (32.6)	46 (33.3)	
3	42 (12.9)	19 (13.8)	
Stent type, n (%)			0.157
Sirolimus-eluting	213 (65.5)	87 (63.0)	
Paclitaxel-eluting	53 (16.3)	32 (23.2)	
> 1 type	59 (18.2)	19 (13.8)	
Stent number, n (%)			0.785
1	120 (36.9)	47 (34.1)	
2–3	143 (44.0)	67 (48.6)	
4–5	52 (16.0)	19 (13.8)	
≥ 6	10 (3.1)	5 (3.6)	
Total stent length, n (%)			0.407
< 20 mm	49 (15.1)	21 (15.2)	
20–60 mm	165 (50.8)	69 (50.0)	
60–100 mm	73 (22.5)	38 (27.5)	
≥ 100 mm	38 (11.7)	10 (7.2)	
Stent diameter, n (%)			0.437
< 3 mm	134 (41.2)	63 (45.7)	
≥ 3 mm	191 (58.8)	75 (54.3)	
Medications			
Aspirin, n (%)			1
No	1 (0.3)	1 (0.7)	
Yes	324 (99.7)	137 (99.3)	
Clopidogrel, n (%)			0.235
No	10 (3.1)	1 (0.7)	
Yes	315 (96.9)	137 (99.3)	
Statins, n (%)			0.224
No	20 (6.2)	4 (2.9)	
Yes	305 (93.8)	134 (97.1)	
β-receptor blockers, n (%)			1
No	89 (27.4)	38 (27.5)	
Yes	236 (72.6)	100 (72.5)	
ACEIs/ARBs, n (%)			0.721
No	162 (49.8)	72 (52.2)	
Yes	163 (50.2)	66 (47.8)	
Calcium channel blockers, n (%)			0.593
No	254 (78.2)	104 (75.4)	
Yes	71 (21.8)	34 (24.6)	

ACEIs, Angiotensin-converting enzyme inhibitors; ARB, angiotensin receptor blockers; BP, blood pressure; LM, left main stem; LAD, left anterior descending artery; LCX, left circumflex coronary artery; LDL-C, low-density lipoprotein-cholesterol; NSTE-ACS, non-ST elevation acute coronary syndromes; OMI, old myocardial infarction; PCI, percutaneous coronary intervention; RCA, right coronary artery; STEMI, ST-elevation myocardial infarction; SA, stable angina

### Predictors of ISR

**Fig. 2:**
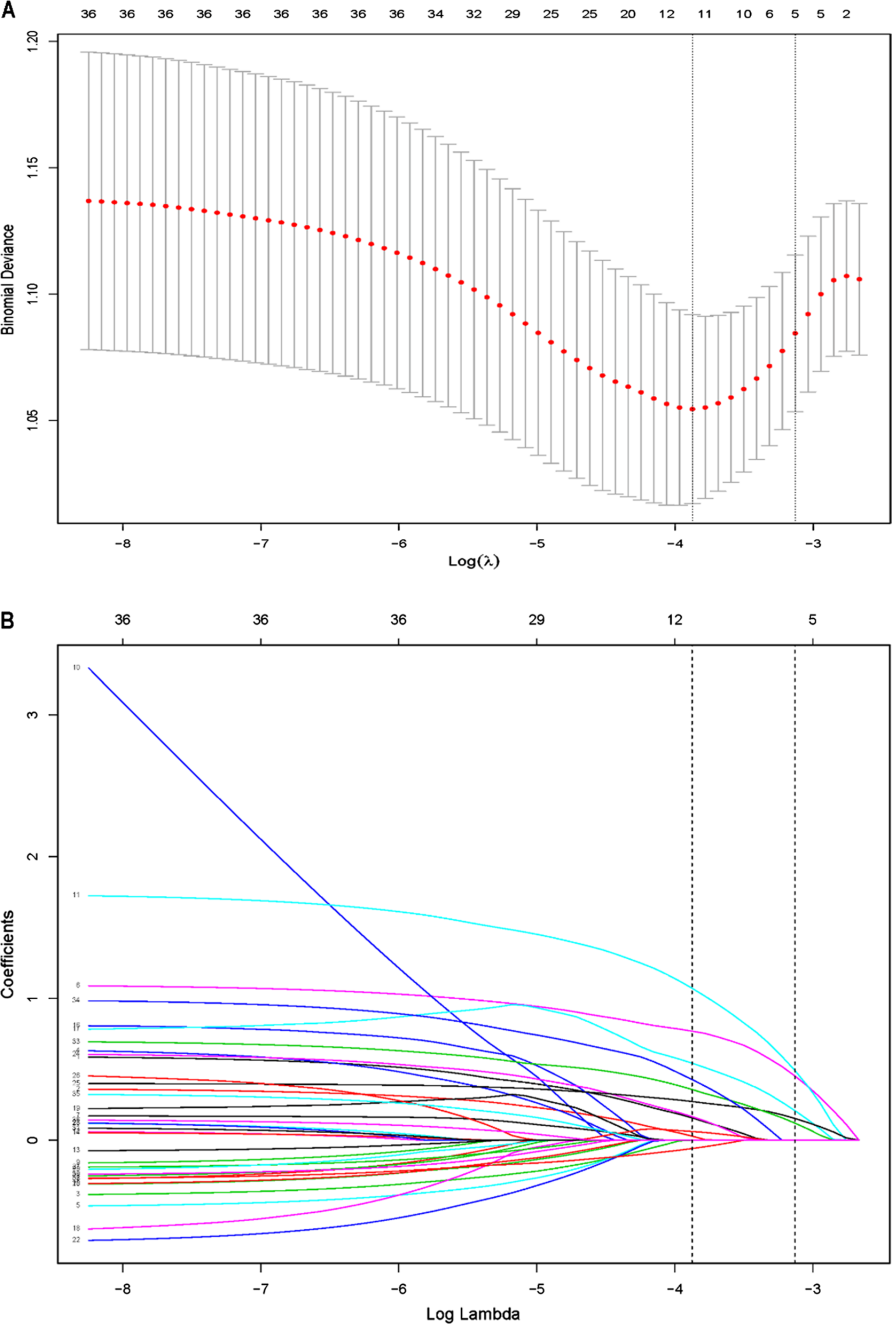
Risk factor selection using the LASSO regression model. *Notes*: **a** Optimal parameter (lambda) selection in the lasso model with the 1 SE of the minimum criteria (the 1-SE criteria). Dotted vertical lines were drawn at the optimal values by using the minimum criteria and the 1-SE criteria. **b** LASSO coefficient profiles of the 36 features. A coefficient profile plot was produced against the log (lambda) sequence. Vertical line was drawn at the value selected, where optimal lambda resulted in five features with nonzero coefficients

Thirty-six variables were reduced to five potential predictors on the basis of 463 patients with the 1-SE of the minimum criteria and nonzero coefficients in the lasso-penalized regression model (Fig. 2a and b). These predictors associated with the ISR of patients included the history of prior PCI, glycemia, stent in LAD, the type of stent, and absence of clopidogrel (Table 2).

**Table 2:** Prediction factors for ISR

Intercept and variable	β	Odds ratio (95% CI)	P-value
Intercept	− 2.656	0.070 (0.030–0.149)	< 0.001
Prior PCI	1.356	3.881 (1.656–9.131)	0.002
Glycemia	1.533	4.634 (1.087–20.965)	0.037
Stent in LAD	1.037	2.821 (1.377–6.260)	0.007
Stent type	1.041	2.832 (1.466–5.452)	0.002
Absence of Clopidogrel	1.762	5.821 (1.463–25.669)	0.013

β is the regression coefficient

CI, Confidence interval; LAD, left anterior descending artery; PCI, percutaneous coronary intervention

### Construction of nomogram

**Fig. 3:**
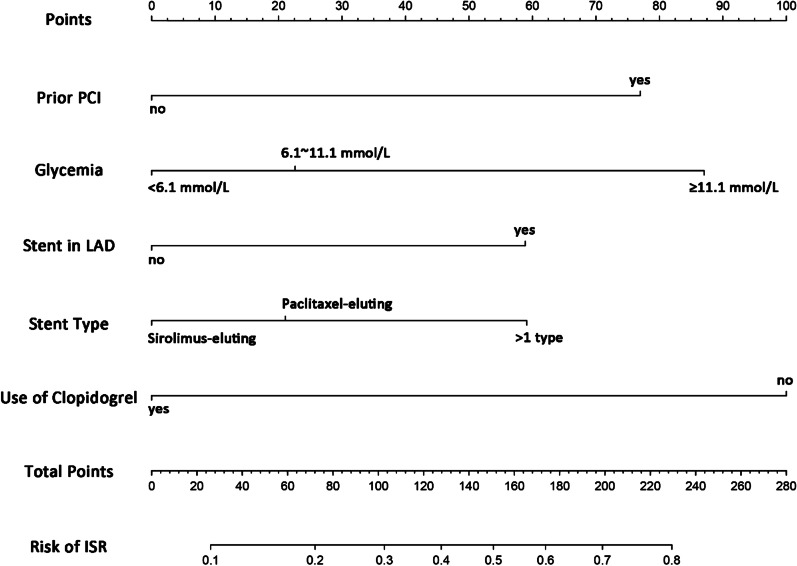
Nomogram to predict the probability of ISR in the patient with stent implantation. *Note*: The risk prediction nomogram was developed in the development set, with prior PCI, glycemia, stent in LAD, stent type and use of clopidogrel

Features screened from the lasso-penalized regression analysis were included in the binary multivariate logistic regression in the development set. The five factors of prior PCI, glycemia, stents in LAD, the type of stent, and absence of clopidogrel were independent risk factors of ISR (Table 2) (all *P* < 0.05). Collinearity diagnostic test did not indicate significant collinearity between independent variables in the regression model, and the variance inflation factors (VIFs) were 1.032, 1.016, 1.063, 1.053 and 1.013, respectively (all VIFs < 10). We then established an individualized nomogram that incorporated the five significant predictive factors based on the logistic multivariate regression analysis (Fig. 3).

### Validation of nomogram

**Fig. 4:**
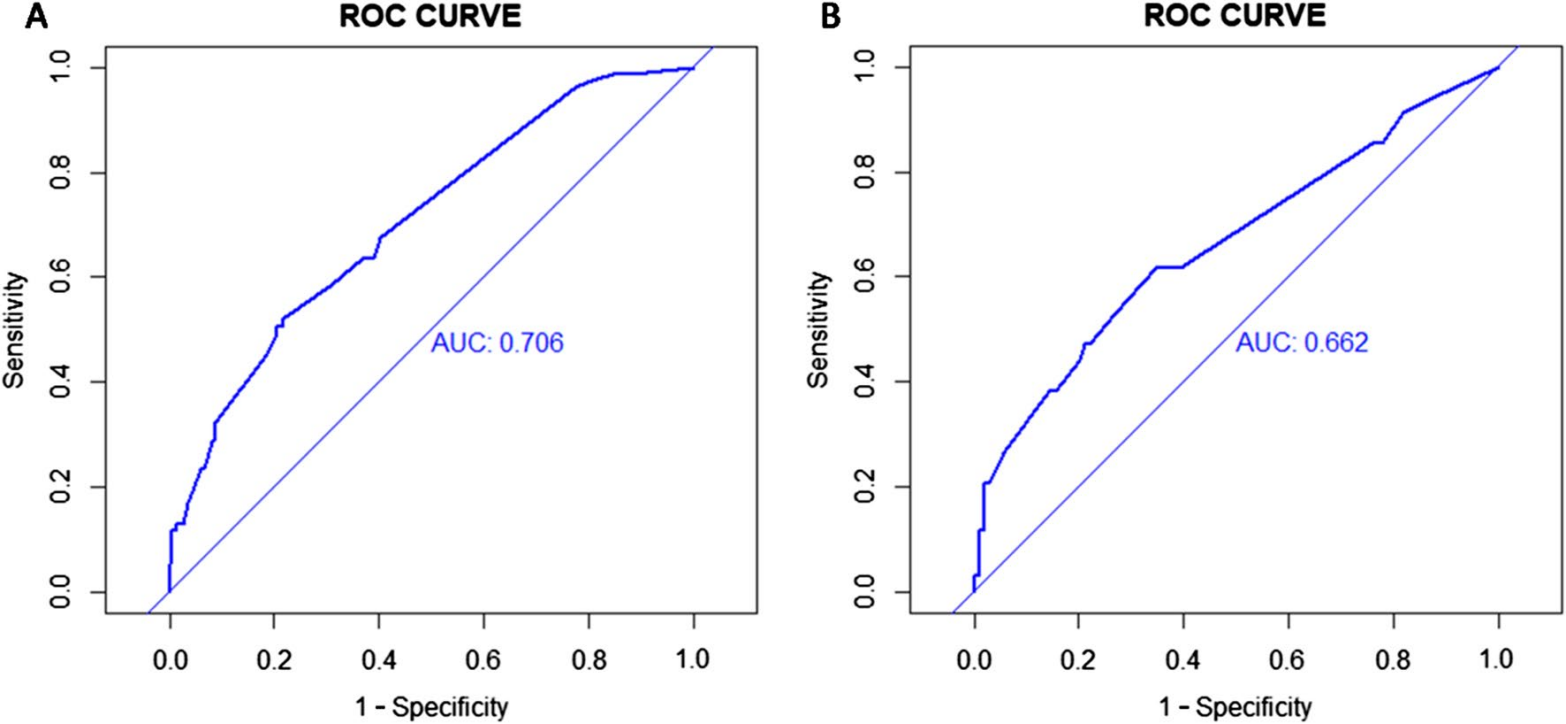
ROC curves for validating the discrimination power of the nomogram. *Note*:** a** Development set. b Validation set (AUC = 0.706 vs. 0.662)

**Fig. 5:**
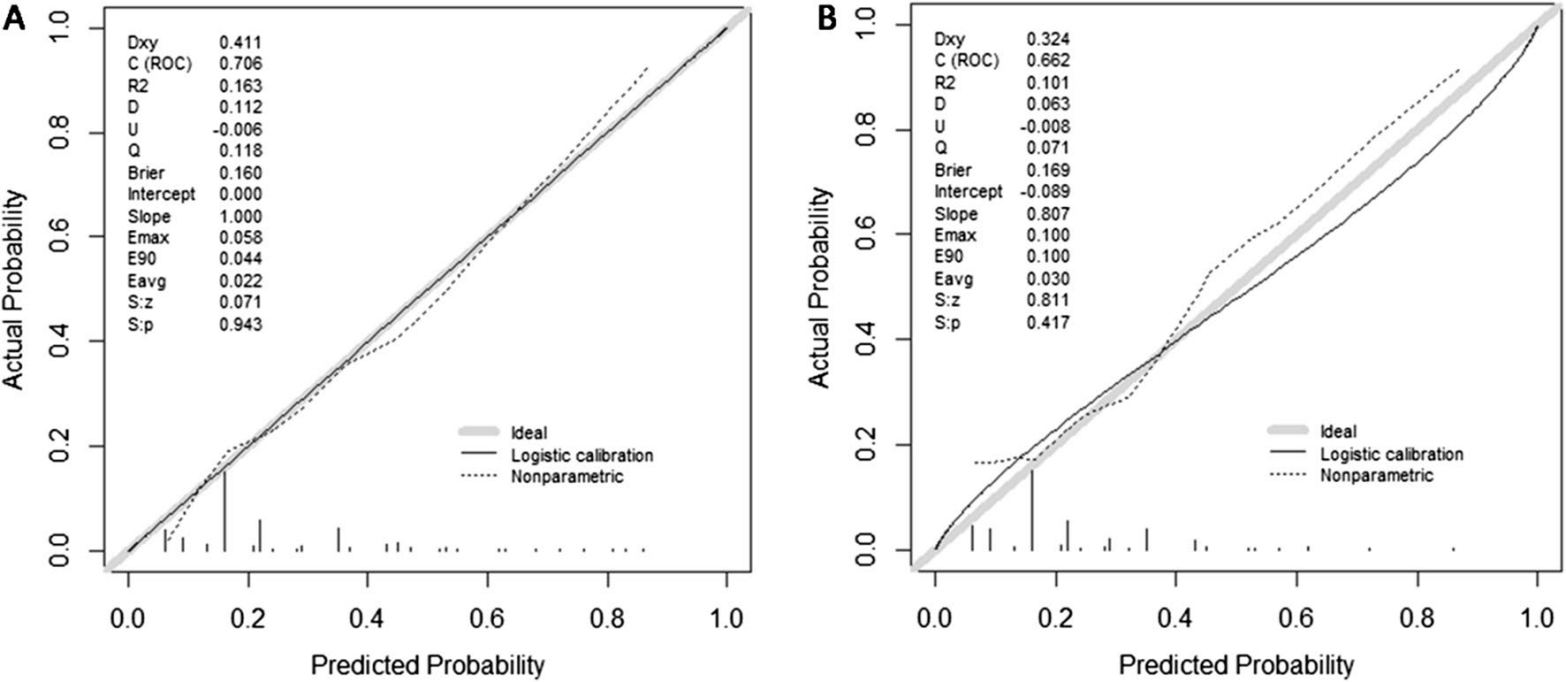
Calibration plots of the nomogram for the probability of PCI patients with in-stent restenosis in the development set and validation set. **a** Development set. **b** Validation set. (All *P* > 0.05)

The ISR nomogram was assessed for internal validation by measuring discrimination, calibration, and clinical usefulness in the development and validation sets. The AUC associated with the ISR nomogram in the development set was 0.706 and was confirmed to be 0.662 in the validation set (Fig. 4a and b), indicating the nomogram prediction model has moderate discrimination. Meanwhile, high quality of calibration plots in both datasets showed that the nomogram model had strong concordance performance compared with an ideal model (*P* = 0.943, and *P* = 0.417, respectively) (Fig. 5a and b), which suggested no significant deviation between predicted and actual probability in both sets.

**Fig. 6:**
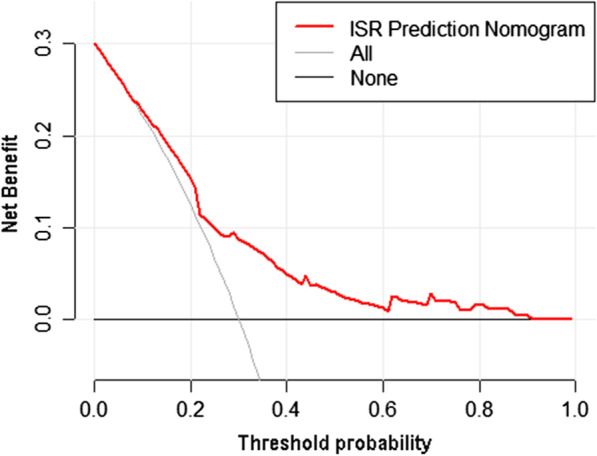
Decision curve analysis for the ISR prediction nomogram in the development set. *Notes*: The y-axis measures the net benefit. The red line represents the ISR risk nomogram. The thin solid line represents the assumption that all patients suffer from ISR. The thick solid line represents the assumption that no patients suffer from ISR. The decision curve analysis indicated that using this ISR prediction nomogram could gain net benefit when the threshold probabilities > 5%

The decision curve analysis for the ISR nomogram is presented in Fig. 6. The results indicated that using the nomogram to predict ISR could acquire much more benefit if the threshold probability is > 9%. Therefore, it could be used to predict the risk of ISR in patients undergoing PCI with high accuracy and a wider range of threshold probabilities, and it might have potentially great significance in clinical application.

## Discussion

Stent implantation is an effective therapy for coronary artery disease. However, ISR has always been one of the most common complications, even in the era of DES. Thus, establishing a predication model of ISR for risk-tailored screening and preventive measure implementation may be pivotal to improve clinical outcomes of patients undergoing PCI. In the present study, five clinical and angiographic characteristics including the history of prior PCI, glycemia, stents in LAD, the type of stent, and absence of clopidogrel were found to independently predict ISR in DES recipients. Moreover, the nomogram prediction model based on these independent factors was constructed and validated, which could provide clinicians with an easy-to-use clinical tool for individualized assessments of patients with high-risk of ISR. Notably, visually and prospectively informing patients of the benefits of risk factor control may improve the patient's understanding of treatment and compliance of therapies, which has great significance for reducing the risk of ISR after stent implantation.

Patients undergoing PCI are exposed to an increased risk of ischemic and bleeding events. Risk models to predict the risk of bleeding events, ischemic events and mortality after PCI have been widely reported. The PRAISE score was a machine learning-based model for the prediction of all-cause death, myocardial infarction, and major bleeding in patients after an acute coronary syndrome [17]. The leading predictors of both myocardial infarction and major bleeding in this model was hemoglobin level, age, left ventricular ejection fraction (LVEF), and estimated glomerular filtration rate (EGFR). The ARC-HBR score is a criterion used for identifying PCI patients with high risk of bleeding events. Risk factors in this criterion includes age, oral anticoagulation, chronic kidney disease, hemoglobin, prior bleeding, thrombocytopenia, chronic bleeding diathesis, liver cirrhosis with portal hypertension, use of NSAIDs or Steroids, active malignancy, prior stroke, and surgery or trauma within 30 days [18]. The PRECISE-DAPT score was also a model to predict bleeding risks for patients after coronary stenting [19], which was based on five factors: integrating age, hemoglobin, white-blood-cell count, creatinine clearance, and prior bleeding. Age, hemoglobin and renal function are the common risk factors in these models. However, these factors were not found in our models for the prediction of ISR in DES, revealing that ISR was associated with different mechanisms, compared with bleeding events, myocardial infarction or death.

Although the exact mechanism of ISR in DES is unclear and probably multifactorial, it is currently accepted that factors including biological, mechanical, and technical issues can facilitate the adverse neointimal hyperplasia and contribute to ISR after stent implantation [2]. Several studies have attempted to identify the independent predictors of ISR. Stolker et al. [20] developed a risk model for predicting restenosis of DES from the EVENT registry and identified age < 60, prior PCI, unprotected left main PCI, saphenous vein graft PCI, minimum stent diameter ≤ 2.5 mm, and total stent length ≥ 40 mm as the predictors of ISR. In another study evaluating the incidence and predictors of target vessel revascularization among 27,107 patients undergoing implantation of BMS or DES, significant predictors of restenosis included prior PCI, emergency or salvage PCI, prior coronary artery bypass grafting (CABG), peripheral vascular disease, diabetes mellitus (DM), and angiographic characteristics [8]. Lately, Zheng et al. [21] analyzed 944 stented lesions from 394 patients with 2nd-generation DES implantation. Factors including DM, previous PCI, postprocedural diameter stenosis and CRP levels were found to independently predict target lesion revascularization. A large patient data pooled analysis from 6 prospective and randomized trials, which included 10,072 patients undergoing DES implantation, suggested that vessel diameter, DM, prior CABG, and prior PCI were patient- and lesion-related predictors of target lesion failure [22].

Individual predictors likely vary between different studies on account of difference in the complexity of patients and candidate variables. However, the overlap in predictive factors, such as prior PCI, prior CABG, and DM, are strongly interlinked with accelerated ISR and repeated target lesion revascularization. Similar to those found in previous studies identifying predictors of ISR, our study also indicates that patient populations with prior PCI and history of DM are prone to ISR.

A history of PCI, which was a consistent and independent predictor of ISR, is closely related to the primary risk factors of atherosclerosis and represents the overall risk of severe coronary lesions requiring further intervention. It is also reported that repeated revascularization is more likely to occur for culprit lesions at a site of previous restenosis [9]. As for DM, patients have a higher risk of developing ISR due to the higher inflammatory response, endothelial dysfunction, platelet hyperreactivity and more aggressive neointimal hyperplasia accompanied by elevated plasma glucose levels [23, 24]. Generally, DM is associated with complicated coronary artery disease characterized by multivessel lesions and diffuse lesions in small vessels, which requires multiple stents with small diameter during PCI. Thus, it can explain at least partly why variables like the total length and minimum diameter of stents were not included in the prediction model in this study. In addition, a noteworthy finding of our study is that uncontrolled glycemia in patients with DM has more predictive value for ISR rather than DM itself. Hyperglycemia itself is reported to be the major cause of diabetic angiopathy [25]. High glucose could increase the expression of monocyte chemoattractant protein-1 (MCP1) and vascular cell adhesion molecule-1 (VCAM-1), thus enhance the monocyte-endothelial cell interaction and promote the atherogenic process and endothelial dysfunction [25]. Endothelial dysfunction is associated with smooth muscle cell proliferation after vascular injury, e.g., restenosis after PCI [26]. Moreover, the formations of advanced glycation end-products (AGE) and AGE-modified low-density lipoproteins (AGE-LDL), which are associated with hyperglycemia, can directly affect the cells of the vascular wall, through mechanisms including the upregulation of MCP1 in both vascular smooth muscle cells and endothelial cells [27, 28]. In addition, Marfella et al. [29] have found that hyperglycemia could increase oxidative damage and may reduce the regenerative potential of ischemic myocardium, while tight glycemic control showed protective effects.

Although several factors associated with ISR in our study are concordant with previous findings, some key predictors including stents in LAD, type of stent, and absence of clopidogrel have not been reported consistently in literature. Most studies suggested that coronary artery intervention restenosis was more frequent for lesions in the LAD than other native coronary arteries, confirming that the LAD may be another potential risk factor for ISR [30–32]. However, different opinions have also been proposed. To the contrary, lesions located in the LAD were reported to have a decreased restenosis rate [33, 34]. In fact, in the present study the results observed after stent implantation for LAD lesions were very similar to those observed in most studies and we believe that stents located in the LAD were associated with an elevated incidence of ISR. For lesions in LAD and other complicated lesions, intravascular ultrasound (IVUS), optical coherence tomography (OCT) and other coronary imaging techniques are recommended for optimizing the treatment strategy. In addition, our findings figured out that sirolimus-eluting stent (SES) was associated with a lower risk of ISR than paclitaxel-eluting stent (PES). Sirolimus and its analogs have a cytostatic effect on coronary artery endothelial cells, while paclitaxel has a cytotoxic effect. Several studies have also indicated that use of SES has a less late luminal loss [7, 35, 36] and a lower rate of late stent thrombosis [37], as compared with use of PES, suggesting a better performance of SES in reducing restenosis. Finally, drugs and polymers of DES can inhibit the excessive neointimal hyperplasia. However, it delays the repair of endothelial cells. Therefore, antiplatelet drugs are still the cornerstone in the treatment of coronary heart disease, especially after PCI. Gianluca et al. [38] investigated the clinical outcome of patients undergoing PCI for ISR with short (6 months) or long (24 months) dual antiplatelet therapy (DAPT). The main findings of this study were that patients receiving revascularization for ISR may benefit from long-term administration of aspirin plus clopidogrel. Similarly, our study showed that the absence of clopidogrel increased the risk of ISR after PCI, suggesting the benefit of appropriately prolonged DAPT duration for patients with high risk of ISR after DES implantation.

Predictor identification and risk assessment are essential and important to an effective medical decision making for preventing restenosis. Multifactorial intervention has shown remarkable benefits on the risks of major cardiovascular events and mortality in patients with diabetic kidney disease [39]. It is expectable that treatments targeting related risk factors will help to lower the incidence of ISR in patients receiving PCI. However, the levels of prognostic utility of prediction models of ISR in prior studies remained less than totally satisfying with c-statistic below 0.7 [8, 9, 20, 40]. In the present study, the best c-statistic derived from the nomogram model in the development set was 0.706 and was confirmed to be 0.662 in the validation set as well, suggesting that the distinct predictors improved the overall discrimination of the models. Moreover, calibration plots and decision curve analysis for the nomogram-based predication model were also performed well, making our findings more convincing and providing broad applicability in clinical practice.

## Limitations

There are several limitations in this study. First, the conclusions drawn from this study are limited by the relatively small sample size. Although the Hosmer–Lemeshow goodness of fit tests were performed and indicated evidence of good fit (Chi-squared = 2.7855, *P*-value = 0.7330), a larger-scale study is warranted to further verify our findings. Second, although we applied strict criteria for inclusion and exclusion to truly reflect the actual condition of disease occurrence in the population underwent PCI as much as possible, it was inevitable to suffer potential selection bias in the screening procedure. The patients with asymptomatic ISR for whom angiographic follow-up is not routinely performed may have been missed. Third, the moderate c-statistic (0.706) of the ISR nomogram indicates that this prediction model remains suboptimal using included candidate variables. Some potential variables unmeasured were not thoroughly informed such as stent gap, stent under-expansion, lesion complexity and other procedure details. In addition, as a single-center study, it may not be enough to exam the robustness of the nomogram. Evidence from other centers is required for further external validation and generalizability evaluation.

## Conclusions

Our study has developed and validated a robust and individualized nomogram for predicting the risk of ISR among PCI patients. This nomogram consists of five common clinical and angiographic characteristics that are easy to obtain and offers clinicians a simple-to-use clinical tool with relatively good accuracy for the early identification and screening of high-risk patients for ISR. With an estimate of individual risk, clinicians and patients can pay more attention to early and reasonable interventions for risk factors.

## Data Availability

The datasets analyzed during the current study are available in the Dryad data repository, https://doi.org/10.5061/dryad.13d31.

## Abbreviations

ISR: In-stent restenosis
PCI: Percutaneous coronary intervention
LASSO: Least absolute shrinkage and selection operator
AUC: Area under the curve
ROC: Receiver operating characteristics
DCA: Decision curve analysis
LAD: Left anterior descending artery
LM: Left main stem
LCX: Left circumflex coronary artery
RCA: Right coronary artery
DES: Drug-eluting stent
BMS: Bare-metal stent
BP: Blood pressure
LDL-C: Low-density lipoprotein
NSTE-ACS: Non-ST elevation acute coronary syndromes
OMI: Old myocardial infarction
STEMI: ST-elevation myocardial infarction
SA: Stable angina
ACEIs: Angiotensin-converting enzyme inhibitors
ARBs: Angiotensin receptor blockers
OR: Odds ratio
CI: Confidence interval
NB: Net benefit
SE: Standard error
VIFs: Variance inflation factors
LVEF: Left ventricular ejection fraction
EGFR: Estimated glomerular filtration rate
CABG: Coronary artery bypass grafting
DM: Diabetes mellitus
CRP: C-reactive protein
MCP1: Monocyte chemoattractant protein-1
VCAM-1: Vascular cell adhesion molecule-1
AGE: Advanced glycation end-products
IVUS: Intravascular ultrasound
OCT: Optical coherence tomography
SES: Sirolimus-eluting stent
PES: Paclitaxel-eluting stent
DAPT: Dual antiplatelet therapy
